# Mapping evidence of free maternal healthcare financing and quality of care in sub-Saharan Africa: a systematic scoping review protocol

**DOI:** 10.1186/s12961-019-0495-1

**Published:** 2019-11-27

**Authors:** Monica Ansu-Mensah, Tahiru Mohammed, Roseline H. Udoh, Vitalis Bawontuo, Desmond Kuupiel

**Affiliations:** 1 Faculty of Health and Allied Sciences, Catholic University College of Ghana, Fiapre, Sunyani, Ghana; 2 Research for Sustainable Development Consult, Sunyani, Ghana; 3 Discipline of Public Health Medicine, School of Nursing and Public Health, University of KwaZulu-Natal, Durban, South Africa

**Keywords:** Maternal healthcare, health financing, free maternal healthcare, quality of care, sub-Saharan Africa

## Abstract

**Background:**

Identifying and addressing research gaps on the implementation of free maternal healthcare financing policies and the quality of service delivery in sub-Saharan Africa (SSA) is essential in ensuring continuous patronage of the services by clients and sustainability of the policies to meet the intended goals. The proposed scoping review is aimed at mapping evidence on free maternal healthcare financing and quality of care in SSA.

**Methods:**

Arksey and O’Malley’s framework, Levac and colleagues’ recommendations, and the Joanna Briggs Institute guidelines will be used to guide the proposed study. A complete keyword search for relevant studies presenting evidence on free maternal healthcare financing and perceived quality of care among women in SSA will be performed in EBSCOhost, PubMed, Web of Science, Google Scholar and SCOPUS. Relevant grey literature from university repositories and international organisations such as WHO and government websites, and the reference lists of included studies will be additionally searched. The Preferred Reporting Items for Systematic Reviews and Meta-analysis: Extension for Scoping Review (PRISMA-ScR) will be used to present the results of the proposed scoping review. NVivo version 11 software package will be employed to extract the relevant outcomes from the included studies using content thematic analysis. Quality appraisal of the included studies for this proposed study will be performed utilising the latest mixed methods appraisal tool.

**Discussion:**

It is anticipated that the results of the proposed study will inform future research and reveal evidence-based information to address potential quality of care issues that may arise because of free maternal healthcare policy implementation in some SSA countries. The proposed study will also be useful to other SSA countries planning to implement free maternal health policy, as they will be able to draw useful lessons to guide them through the process.

## Background

Reducing maternal and child mortality remains a global challenge. This challenge received much attention during the launch of the Millennium Development Goals in the year 2000 by the United Nations and partners such as WHO [1–5]. It is estimated that 99% of all maternal-related deaths occur in low- and middle-income countries, of which most (66%) are located in sub-Saharan Africa (SSA) [5–8]. Maternal mortality in SSA is 1 in 39 live births, whereas it is 1 in 3800 live births in high-income countries [9, 10], 1 in every 130 live births in Oceania, and 1 in every 160 live births Southern Asia [5–8]. Therefore, increasing access to quality maternal healthcare services in SSA has been a global priority since the inception of the Millennium Development Goals to date.

To improve access to maternal healthcare services for all women, particularly the poor, various health financing policies, such as free maternal healthcare schemes, aimed at removing financial barriers have been adopted and implemented by some SSA countries. Most of these free maternal healthcare policies cover the WHO’s priority interventions such as antenatal care, delivery and postnatal care, which are recognised by WHO as essential for improving maternal health outcomes [1, 11]. For instance, with the intervention of antenatal care [11] , every pregnant woman is supposed to visit the care provider at least four times during pregnancy for services such as health education, counselling, screening and testing, and treatment, where necessary, with the aim of promoting the health status of both mother and child [9].

Although these free maternal healthcare policies have improved access to healthcare, particularly for pregnant women who are in the low-income brackets, the quality of the service delivery is essential to achieve the aim of the policy [1, 9, 11]. The quality of maternal healthcare services rendered at the various health facilities is particularly crucial to facilitating continuous utilisation of the services by clients, thereby ensuring the desired maternal health outcomes [12–16]. Therefore, the proposed scoping review is aimed at mapping evidence on free maternal healthcare financing and quality of care in SSA focusing on clients, healthcare providers or healthcare professionals, and healthcare managers’ perspectives. We anticipate that the results of this scoping review will reveal useful evidence-based information and identify gaps for future studies to improve maternal healthcare delivery in SSA. We also anticipate that the study findings may influence policy decisions to improve the quality of care as well as contribute to the achievement of United Nations Sustainable Development Goal 3.1, which stipulates that a maternal mortality of less than 70 maternal deaths per 100,000 live births should be achieved by 2030 [17].

## Methods

### Overview

Scoping reviews are considered useful in representing series of literature that exists around a subject of interest and helps to focus the research questions by registering existing research findings and identifying research gaps [4]. A scoping methodology is also considered a useful approach for determining the basic and value of follow-up of a primary study or a full systematic review [5]. Based on this, the current scoping review will be guided by the enhanced version of Arksey and O’Malley’s framework [18], Levac et al.’s recommendations [19] and the 2015 Joanna Briggs Institute guidelines [20] as well as the Preferred Reporting Items for Systematic Reviews and Meta-Analyses Protocol (PRISMA-P) (Additional file 1). The framework involves the following: identifying the research question; identifying relevant studies; study selection; charting the data; and collating, summarising and reporting results [18].

### Identifying the research question

Our research question is: What is the evidence on free maternal healthcare financing and quality of maternal healthcare in SSA?

The sub-review questions will be as follows:

- What is the perspective of clients on the quality of care provided under the free maternal healthcare policy in SSA?
- What is the perspective of healthcare providers on the quality of care provided under the free maternal healthcare policy in SSA?
- What is the perspective of healthcare managers on the quality of care provided under the free maternal healthcare policy in SSA?

The Population, Concept, and Context (PCC) mnemonic [20] used to determine the eligibility of the primary research question for the proposed scoping review is shown in Table 1.

**Table 1:** PCC (Population, Concept, and Context pneumonic) framework for defining the eligibility of the primary question for the scoping review

P-Population	Qualitative, quantitative or mixed methods studies involving free maternal healthcare financing policies: we defined free maternal healthcare policy as any insurance scheme that seeks to eliminate financial barriers associated with the use of maternal and child healthcare services by pregnant and post-natal mothers
C-Concept	Quality of care: quality of maternal healthcare is defined based on client, healthcare provider and health manager perspectives
C-Context	Sub-Saharan Africa: In this study, sub-Saharan Africa will refer to all countries in the WHO African Region [21]

### Identify relevant studies

We will conduct a thorough and complete search of numerous bibliographic databases to include all relevant studies on free maternal healthcare policies and the quality of healthcare delivery in SSA irrespective of publication standing whether published, unpublished or in press. A full search will be conducted in EBSCOhost, PubMed, Web of Science, Google Scholar and SCOPUS for relevant studies. Relevant grey literature from university repositories and international organisations such as WHO and government websites will also be searched. We will additionally explore the reference lists of all included studies for relevant studies. We will use a combination of the following keywords to search for relevant studies from the electronic databases: ‘free maternal healthcare’ ‘healthcare financing’ ‘health insurance scheme’ ‘maternal healthcare’ ‘quality of care’. We will use Boolean terms ‘AND’ and ‘OR’ to separate keywords. We will also include appropriate Medical Subject Heading (MeSH) terms and keywords to identify relevant studies. Study designs will be limited to only qualitative, quantitative or mixed methods studies and the search language limited to English due to a lack of expertise to interpret other languages. However, date of publication limitations will be removed. Table 2 shows a pilot search conducted in PubMed demonstrating the possibility of conducting the proposed scoping review.

**Table 2:** Pilot search in PubMed electronic database

Date	Database	Keywords	Search results
31/08/2019	PubMed	• Line 1: “free maternal healthcare”[All Fields] OR “free healthcare financing”[MeSH Terms] AND • Line 2: “mothers”[MeSH Terms] OR “expectant mothers”[All Fields] OR • Line 3: “pregnant”[All Fields] OR “Pregnancy”[MeSH Terms] OR “post-natal”[All Fields] OR • Line 4: “women”[All Fields] OR “woman”[All Fields] AND “quality”[All Fields] OR • Line 5: “quality of health care”[MeSH Terms] OR “quality of maternal healthcare”[All Fields] OR • Line 6: “quality of care”[All Fields] OR “quality of maternal health service”[All Fields] OR • “quality of maternal health delivery”[All Fields] AND • Line 7: “Africa”[All Fields] OR “sub Saharan Africa”[All Fields] • Line 8: “humans”[MeSH Terms]	64,193

### Eligibility criteria and study selection

To ensure the selection of relevant studies for this review, the study selection will be guided by the eligibility criteria as specified under the inclusion/exclusion criteria.

#### Inclusion criteria

We will include studies that meet the following criteria:

- Evidence of the study conducted in SSA
- Studies presenting evidence of free maternal healthcare policy
- Studies reporting evidence on quality of maternal healthcare services
- Primary studies

#### Exclusion criteria

This study will exclude the following:

- Studies conducted in countries not included in the WHO African Region
- Studies targeting women of fertility age
- Studies reporting evidence on quality of care from the health providers’ perspective
- Studies reporting evidence on quality of care from the funders’ or representatives of health financing institutions’ perspectives
- Other types of reviews

### Study selection

**Fig. 1:**
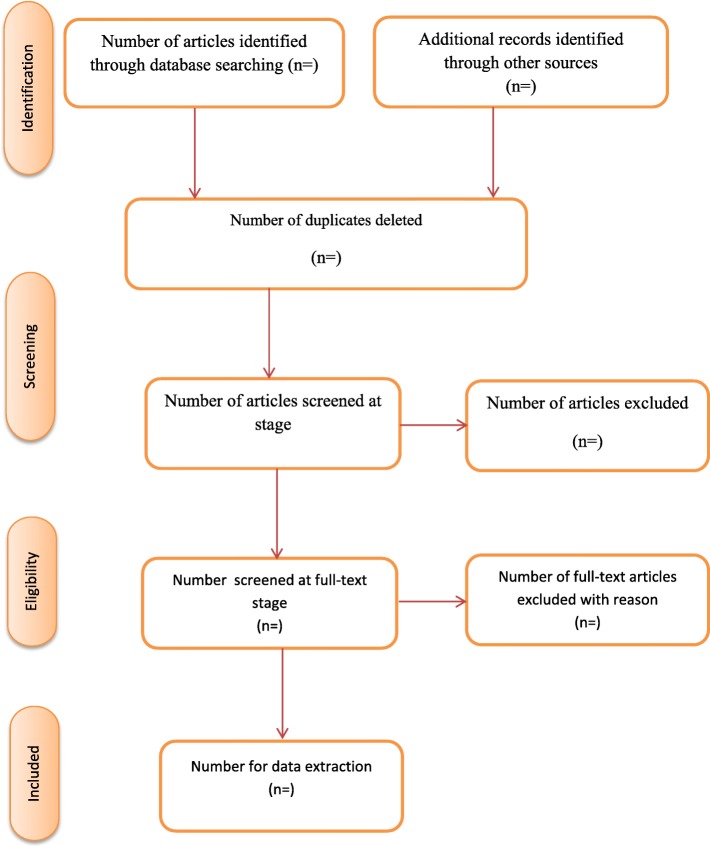
PRISMA 2009 flow diagram [21]

Screening of relevant studies for inclusion in the proposed scoping review will be conducted in three phases. First, the principal investigator will conduct the title screening of retrievable studies from the online database. All eligible studies will be imported onto an endnote library X7 created for the study. At the second (abstract screening) and third (full-text screening) stages, two reviewers will independently sort the studies into two classifications (‘inclusive’ and ‘exclusive’) using the eligibility criteria. Discrepancies between reviewers’ responses at the abstract screening stage will be resolved through a discussion by the review team until a consensus is reached. However, discrepancies in reviewers’ responses at the full-text screening stage will be resolved by involving a third reviewer. In the situation where a full-text article cannot be found from the databases, assistance would be sought from the Catholic University’s library or the full-text will be requested from the authors via email. Inter-rater agreement (Cohen’s kappa co-efficient (k) statistics) between reviewers’ responses will be calculated along with McNemar’s χ^2^ statistics using Stata 14 following full-text screening. The search record will also be adequately documented as follows: date of search, database, keywords, number of retrievable studies and number of eligible studies. We will follow an adapted PRISMA [21] to present the screening results of the proposed scoping review, as shown in Fig. 1.

### Charting the data

A data charting form will be developed for extraction of relevant data from the included studies in the scoping review. This form will include the following: author and date, study title, objective/aim of the study, study design, study setting, type of free maternal healthcare policy and perceived quality of care. The data extraction form will be piloted by two independent reviewers using a random sample of 10% of the included studies to ensure consistency and accuracy. The data extraction form will be adjusted as required based on feedback from the two independent reviewers. We will constantly update the data extraction form to enable adequate abstraction of all relevant data to answer the review question.

### Collating, summarising and reporting the results

We will extract data related to free maternal health policies and perceived quality of care in SSA using content thematic analysis approach [22]. NVivo version 11 will be employed for the content thematic analysis of the included studies and the findings on free maternal health policies and perceived quality of care in SSA summarised and presented using a narrative approach. Emerging themes will also be reported.

### Quality appraisal

To evaluate the quality of the studies that will be included in the proposed scoping review, the Mixed Method Quality Appraisal Tool (MMAT) Version 2018 will be used to evaluate the methodological quality of all the studies included. The relevance of the study, study design, adequacy and methodology, data collection, analysis of data, and study findings will be examined using the MMAT tool. The quality assessment will be helpful in reporting the risk of bias of the studies included. The quality of the included studies will be graded by calculating the total percentage quality score as specified by the 2018 MMAT. A percentage quality score ranging from ≤50% will be considered as low quality, 51–75% will be considered as average quality, and 76–100% will be considered as high quality.

## Discussions

The proposed scoping review will aim to map evidence on free maternal healthcare financing and quality of care in SSA. Improving the quality of care during pregnancy has many benefits such as improved utilisation of antenatal services [23, 24], early detection of infection and prompt linkage to care [25], and reduction of maternal morbidities and mortalities [7], among others. It is anticipated that the results of the proposed study will inform future research and reveal evidence-based information to address the potential quality of care issues that may arise because of free maternal healthcare policy implementation in some SSA countries. The proposed study will also be useful for other SSA countries planning to implement free healthcare financing schemes for maternal health, helping them to draw useful lessons to guide its implementation. The proposed study will thus contribute to healthcare systems strengthening and improve research on free maternal healthcare financing schemes and the quality of care in SSA.

## Conclusion

The results of this systematic scoping review research will help identify gaps useful for future studies, such as systematic reviews, meta-analysis and primary studies, to improve the quality of maternal healthcare delivery services in SSA.

## Supplementary information

**Additional file 1.** PRISMA-P checklist.

## Data Availability

We have duly cited all studies and data is presented in the form of references.

## Abbreviations

MMAT: Mixed Method Quality Appraisal Tool
SSA: sub-Saharan Africa
